# Junior doctors: when fresh blood fast-tracks the fight against COVID-19

**DOI:** 10.1136/postgradmedj-2020-138782

**Published:** 2020-09-03

**Authors:** Elisabeth Ashton, Charbel Skayem, Charles Ouazana-Vedrines, Pierre Hamann

**Affiliations:** Oncology Department, Assistance Publique des Hôpitaux de Paris (APHP), Cochin Hospital, Paris, France; Faculté de Médecine, Université de Paris, Paris, France; Faculté de Médecine, Université de Paris, Paris, France; Hopital Henri Mondor, Assistance Publique des Hopitaux de Paris, Creteil, France; Faculté de Médecine, Université de Paris, Paris, France; Psychiatry Department, HEGP Hospital, AP-HP,Paris, France; Dermatology Department, Institut Gustave-Roussy,Villejuif, France; Faculté de Médecine du Kremlin Bicêtre, Université Paris-Saclay, Paris, France

## INTRODUCTION

On 11 March 2020, the WHO declared COVID-19 as a Public Health Emergency of International Concern. In Europe, particularly in France, hospitals were urged to take prompt measures: cancellation of non-emergent procedures, dedication of units and wards to patients with COVID, and reallocations of medical and non-medical staff.^1^

Paris and its surrounding region, Ile-de-France, were the most severely impacted regions in France. With a total population of 12 million inhabitants (19% of the French population), hospitals had to provide up to 2600 additional beds in intensive care units (ICUs) (100% more than available) to cope with the pandemic. Medical residents in France represent 32% of public hospital physicians. In the Parisian area, there are 5400 residents, all specialities included, distributed in 100 hospitals. Residents were, along with the other healthcare professionals, on the front line of the fight against COVID-19.

Every year, up to 1000 residents are not assigned to any hospital unit, for personal reasons or scientific research projects. At the beginning of the outbreak in early March, identifying this unallocated manpower was essential to alleviate work pressure within hospitals, yet very challenging. Thus, dispatching non-assigned volunteers to departments in need was non-trivial. In France, this mission is usually managed by the Regional Health Agency (Agence Régionale de Santé (ARS)). In the context of the COVID-19 epidemic, ARS and Paris Public Hospitals (Assistance Publique—Hôpitaux de Paris) were overwhelmed by overall health management issues, including addition of hospital beds, supply of personal protective equipment and identification of available non-medical staff.

On 16 March 2020, three residents who had never worked together—one oncologist, one psychiatrist, one dermatologist—formed a crisis management team (CMT). These three volunteers offered their support to the ARS, and they were delegated for this mission.

Their main targets were to list voluntary available residents based on their skills, to identify hospitals requiring human support and to eventually allocate the appropriate human resources in the identified units.

This new self-managed organisation was set up in record time using information communication technology (ICT) tools to dispatch non-assigned volunteers during this unprecedented COVID-19 crisis. Our objective is to highlight efficiency that millennials can bring in times of crisis, by presenting the initiative that these residents took in the most impacted region in France during the COVID-19 outbreak.

## PROCESS

To properly set up the CMT, residents of the CMT needed available manpower for organisation. These volunteers had to be off duty (for pregnancy, medical issues, etc). A call for volunteers to join the CMT was made on social networks. In 24 hours, 31 residents were recruited to form the monitoring team (17 residents) and the matching team (14 residents). A total of 250 residents already working in the Parisian region hospitals were chosen as a local source to evaluate staff needs in each hospital.

Then, the step was to identify available volunteers to be dispatched to different hospitals. Volunteers had to fill out an electronic form, published by the two Paris Residents Unions on their usual social networks and email list. Collected data were as follows: the resident’s speciality, year of speciality, skills (especially in emergency and critical care), availability, location and contacts.

**Figure 1:**
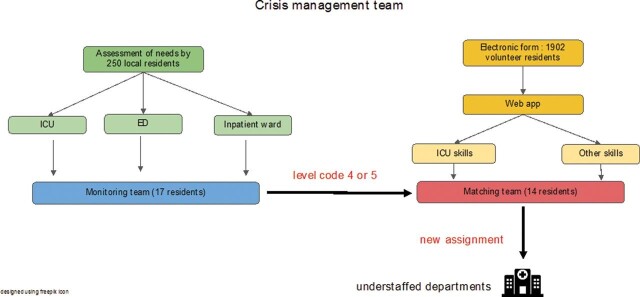
Management of volunteer residents by the crisis team during COVID-19 outbreak in Paris.The monitoring team with the assessment of needs in the hospitals by our local contacts are represented on the left side. The functioning of the matching team and the reassignment of residents in understaffed departments according to the warning signal are represented on the right side. ED, emergency department ICU, intensive care unit.

All information was collated in a web app to sort, organise and display the data. This app was developed within 48 hours by a computer engineer, a close relative of one of the crisis team founders (figure 1).

Departments in need had to be identified. For this, one or two residents, as local contact, were assigned by the CMT in each ICU, emergency department and inpatient ward. Their role was to identify the local problems faced by the residents, especially short staffing. Daily updates were provided to the CMT by using social networks.

Each situation was classified by the monitoring team using an alert level code ranging from 1 (suitable number of residents) to 5 (understaffing requiring immediate action). Levels 4 and 5 triggered the workforce search process. Daily updates were provided to the CMT.

Consequently, volunteers were dispatched by the matching team to the understaffed departments, according to their location and skills. Matching also took into consideration general conditions like housing, transportation, insurance coverage and wages. Then, two weekly follow-ups of the newly assigned resident were made to.

After reassignment of the residents, the monitoring team made sure to follow up on their mental well-being and integration in the new departments. Volunteering psychiatry residents provided urgent moral support for all residents in distress.

From 16 March until 10 May, a total of 1902 residents responded, of which 1203 volunteers within the first 48 hours. A total of 578 volunteers were dispatched between 22 March and 10 May: 230 in ICUs, 93 in emergency departments and 255 in inpatient wards. Approximately 85% of the volunteers were dispatched before 10h April, the peak of the epidemic in Ile-de-France. After 2 weeks of matching, all the available volunteers with intensive care competencies were reallocated with 30 residents from other regions of the country.

Deploying residents to a different department of their hospital or to a new hospital was not easy. Amending some regulations was indispensable. The CMT decided to bring up their suggestion to higher authorities in the Ministry of Health and the Ministry of Education, with the help of the two Paris Residents Unions, and they succeeded.^2^

## BEHIND THE RESIDENTS’ INITIATIVE

While millennials have a bad reputation in the medical circles, the traits that sometimes chafe their older colleagues may make the profession healthier and more technologically savvy.

This was clearly highlighted in the COVID-19 pandemic in France whereby the CMT was able to overcome a major obstacle in controlling the outbreak in the Parisian region and its surrounding, which were the most impacted by the pandemic. Being digital natives, they are used to having information at their fingertips and having constant connection with peers.^3^

Speed and acceleration were the main characteristics of the residents’ approach. This was made possible because of the fast pace in problem solutions that technology adapted them to. Globalisation and rapid advancements in technology require different communication and professional engagement styles, attitudes toward diversity and idealism about the future, which were well projected by the CMT team.^4^

Their capacity in fast networking helped them achieve a mission they were not in charge of. On the other hand, physicians from older generations, who tend to be less experienced in social media and technology, were unable to initiate a step to overcome the human resource obstacle. This reflects that millennial residents are responsible adults, who are showing leadership. They have begun to enter the physician workforce, with a vision of building a promising future for the medical field.^5^

## CONCLUSION

The COVID-19 pandemic has revealed the virtues of devotion and commitment that drove medical residents throughout their long curriculum. The crisis team has reflected the potential of millennial junior doctors to take lead in times of crisis. This self-managed organisation provided a reliable and agile answer to the staffing problem within hospitals, thanks to their familiarity with social networks and ICT tools. While COVID-19 revealed the responsible identity of future doctors, it has also disclosed current fundamental flaws in the healthcare system. Will this pandemic be the trigger to finally reshape health and development institutions?
